# Pupillometry pain index decreases intraoperative sufentanyl administration in cardiac surgery: a prospective randomized study

**DOI:** 10.1038/s41598-020-78221-5

**Published:** 2020-12-03

**Authors:** Vivien Berthoud, Maxime Nguyen, Anouck Appriou, Omar Ellouze, Mohamed Radhouani, Tiberiu Constandache, Sandrine Grosjean, Bastien Durand, Isabelle Gounot, Pierre-Alain Bahr, Audrey Martin, Nicolas Nowobilski, Belaid Bouhemad, Pierre-Grégoire Guinot

**Affiliations:** 1 Anaesthesiology and Critical Care Department, Dijon University Hospital, 2 Bd Maréchal de Lattre de Tassigny, 21000 Dijon, France; 2 LNC UMR1231, University of Burgundy Franche-Comté, 21000 Dijon, France

**Keywords:** Medical research, Pain management

## Abstract

Pupillometry has proven effective for the monitoring of intraoperative analgesia in non-cardiac surgery. We performed a prospective randomized study to evaluate the impact of an analgesia-guided pupillometry algorithm on the consumption of sufentanyl during cardiac surgery. Fifty patients were included prior to surgery. General anesthesia was standardized with propofol and target-controlled infusions of sufentanyl. The standard group consisted of sufentanyl target infusion left to the discretion of the anesthesiologist. The intervention group consisted of sufentanyl target infusion based on the pupillary pain index. The primary outcome was the total intraoperative sufentanyl dose. The total dose of sufentanyl was lower in the intervention group than in the control group and (55.8 µg [39.7–95.2] vs 83.9 µg [64.1–107.0], p = 0.04). During the postoperative course, the cumulative doses of morphine (mg) were not significantly different between groups (23 mg [15–53] vs 24 mg [17–46]; p = 0.95). We found no significant differences in chronic pain at 3 months between the 2 groups (0 (0%) vs 2 (9.5%) p = 0.49). Overall, the algorithm based on the pupillometry pain index decreased the dose of sufentanyl infused during cardiac surgery.

**Clinical trial number: **NCT03864016.

## Introduction

Since the 1960s, the systematic administration of opioids has been one of the pillars of modern general anaesthesia. Opioids used during anaesthesia are synthetic derivatives characterized by their extreme power and short duration of action^1^. In cardiac surgery, opioids have antinociceptive and cardioprotective effects that have led to their widespread use^2^. The effects of opioids are highly variable between subjects, and cardiopulmonary bypass (CPB) surgery changes the pharmacokinetics of opioids^3^. Moreover, these drugs are associated with side effects like respiratory depression or hyperalgesia^4, 5^. To counter this problem, several strategies based on assessment of nociceptive/antinociceptive balance or limiting opioid use have been developed and studied^6, 7^.

It is recognized that clinical parameters such as heart rate or blood pressure are insufficient to track nociception/antinociception balance during anesthesia, particularly in cardiac surgery patients undergoing CPB, and several analgesia monitoring devices have therefore been developed^8^. Among these strategies, the assessment of pupillary dilation can reflect the response to stimuli in patients during surgery or in the ICU^9–11^. Authors have demonstrated that the pupillary dilatation reflex (PDR) can be monitored during surgery in order to tailor opioid administration to operative nociception stimuli^12^. In this setting, pupillometry appears to be a promising tool to decrease the use of opioids during surgery^13^. However, because PDR assessment can be a subjective process, a novel index [pupillary pain index (PPI)] based on pupillary diameter variation in response to electrical stimuli has been developed^14^. By grading pupillary dilatation with a standardized stimulus, the PPI offers a nociceptive scale. A study demonstrated that the PPI can assess the level of analgesia during anesthesia^15^. To date, no study has evaluated the ability of the PPI to guide opioid use during cardiac surgery with cardiopulmonary bypass.

The objective of the present study was to determine whether the pupillary pain index could be used to decrease operative opioid use by assessing the nocipetion/antinociception balance. Our hypothesis was that the use of an algorithm for sufentanyl administration based on the PPI would decrease the total dose administered during anesthesia.

## Materials and methods

The study objectives and procedures were approved by an independent ethics committee (*Comité de Protection des Personnes, Ile de France IV* No. 2018-A03102-53, chairman Dr Shahnaz KLOUCHE, approved 18 January 2019). The manuscript conforms to CONSORT 2010 guidelines. All patients received written information about the study and gave their written consent to participate prior to surgery. Informed consent was obtained from all subjects. The study was recorded at ClinicalTrials.gov (Identifier: NCT03864016, March 6 2019). We conducted a prospective, open-label, randomized, controlled, parallel-arm, monocentric clinical trial from March 2019 to October 2019. This study was carried out in accordance with guidelines for Good Clinical Practice and the Declaration of Helsinki. The protocol study is available as Supplementary Information.

### Participants

Inclusion criteria were as followed: patients over 18 years old, American Society Anesthesiologists score (ASA) lower than 4, elective cardiac surgery with CPB (coronary artery bypass graft surgery with or without associated valvular surgery). We excluded patients with preoperative cognitive dysfunction (Mini-mental State < 13), chronic opioid use, known opioid intolerance, ophthalmologic disease (ocular disease, corneal lesion or contact lens wearer, neurological factors that can influence the pupillary reflex), non-affiliated to national health insurance, pregnant women, and emergency surgery.

### Intervention

**Figure 1:**
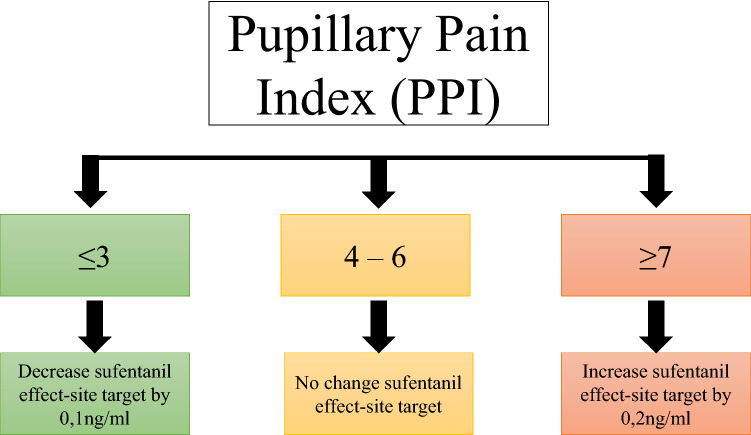
Pupillary Pain Index algorithm.

The intervention group received PPI-based sufentanyl administration. Sufentanyl effect-site target concentration (Ce) was set at 0.3 ng/ml then the target concentration was adapted to the PPI score using a predefined algorithm (Fig. 1, Supplementary Information). The PPI was obtained in a standardized manner: 2 min before orotracheal intubation (T1) 2 min before skin incision (T2); after sternotomy (T3); at the start of CPB (T4); at CPB weaning (T5); at skin closure (T6). The PPI was systematically checked after each sufentanyl dose change. The anesthetist monitored the PPI at each hemodynamic change to assess the relation with nociceptive stimulus. In the case of arterial hypertension with a low PPI, an injection of intravenous urapidil (5 mg) was performed. This injection could be repeated (maximal dose of 40 mg) until adequate blood pressure management (TAS < 130 mmHg). In case of arterial hypertension with high PPI, the sufentanyl dose was increased by step of 0.2 ng/ml.

In the PPI group, the pupillometry was monitored with the AlgiScan video pupillometer (IDMED, Marseille, France). The pupillometer detects the pupil with an infrared camera, so the measurement is non-invasive and the device never comes into contact with the eye. The AlgiScan PPI mode is programmed to deliver electrical stimulation of increasing intensity from 10 to 60 mA by steps of 10 mA^14^. The pupillary diameter is measured during electrical stimulation. When dilation reaches a threshold of 13% change compared to the initial size, the electrical stimulation stops. The intensity of the stimulation that triggered the 13% dilation is used to calculate the PPI score (range from 1 to 9)^14^.

The standard group received conventional sufentanyl administration. Sufentanyl effect-site target concentration (Ce) was set at 0.3 ng/ml, and the target concentration was adjusted by the attending physician according to usual practice (standard care). To avoid bias, the attending physician was not informed of the patient participation in the study.

### Outcomes

#### Primary outcome

The main outcome was the total sufentanyl (µg) dose administered during anesthesia.

#### Secondary outcomes

The secondary outcomes were the cumulative doses of morphine 48 h after the surgery, static and dynamic VAS at the different time points (at extubation [H0], H6, H24, and H48), peak troponin (H6) and chronic pain assessed with the Neuropathic Pain Diagnostic Questionnaire (DN4) three months after surgery. Hospital and ICU length of stay were also collected. ICU and hospital discharge were not defined by protocol and were at the discretion of the treating physicians.

### Operative management

Preoperative, operative and postoperative care were standardized for all patients. Premedication (alprazolam) was administered at the patient’s request on the day before and the day of surgery. Cardiopulmonary bypass management was standardized for all patients as previously described^16–18^. Patient monitoring during anesthesia comprised continuous measurement of invasive blood, heart rate, oxygen saturation (SpO_2_), central venous pressure, hypnosis (bispectral analysis (BIS XP monitor, Medtronic, France)), bladder temperature, diuresis, neuromuscular monitoring (NMT), and inspired/expired fraction of carbon dioxide and oxygen. Anesthesia induction was performed with propofol (Schnider model) and sufentanyl (Gepts model) administered by effect-site target-controlled infusion (Base Primea; Fresenius-Kabi, Germany)^16^. Propofol initial effect-site target concentration (Ce) was set at 4 μg/ml. All patients received intravenous cisatracurium (0.15 mg/kg) and intravenous ketamine (50 mg) at anesthesia induction. Intubation was performed when the NMT was equal to zero. Mechanical ventilation was performed with a tidal volume between 6 and 8 ml/kg, and a FIO_2_ between 40 and 60%. The objective of end tidal carbon dioxide tension was between 35 and 45 mmHg. Intravenous administration of cisatracurium was based on NMT monitoring, and propofol was administered in target-site effect concentration to obtain a BIS between 40 and 60 throughout the surgery, and to avoid burst suppression.

After surgery, sedation and mechanical ventilation were continued for all patients until haemodynamic stability, normothermia, and absence of significant active haemorrhage (less than 1 ml/kg/h) could be verified. Sedation was maintained between − 2 and − 3 on the Richmond Agitation-Sedation Scale (RASS). The patients were managed by a team of physicians specialized in the postoperative care of cardiac surgery patients, including a cardiologist. Circulatory support was guided by institutional protocols to achieve predefined endpoints: mean arterial pressure > 65 mmHg, cardiac index > 2.2 l/min/m^2^, and urine output > 0.5 ml/kg/h, as previously described^17^. Extubation was standardized according to institutional protocol^17^.

### Analgesia

Analgesia was standardized. At the end of the surgery, all patients received a presternal catheter (Halyard ON-Q, Alpharetta, USA) which was placed by the surgeon. A bolus of 10 ml of levobupivacaine (1.25 mg/ml) was performed. Then the catheter was connected to a pump with a flow of 8 ml/h of levobupivacaine (1.25 mg/ml) during the 48 first post-operative hours^19^. All patients had administration of 1 g of intravenous paracetamol every 6 h on day 0, with a switch to oral form at day 1. All patients had intravenous morphine titration (bolus between 2 and 3 mg every 7 min) until pain was below 3 on the visual analogue scale (VAS). Then, morphine was administered by patient-controlled analgesia (PCA) with the following parameters: bolus of 1 mg, refractory period of 7 min, maximal dose of 20 mg every 4 h^19^. The use of other analgesia drugs (nefopam, ketoprofen, and tramadol) was left to the discretion of the attending physician.

### Data

All data were continuously recorded on a case report form by a clinical data manager who was blinded to patient allocation. The following perioperative variables were recorded: age, gender, body weight, height, personal medical history, ASA score, EuroSCORE II, type of cardiac surgery, preoperative left ventricular ejection fraction, the duration of CPB, duration of aortic clamping, total sufentanyl dose (µg), total propofol dose (mg), need for intraoperative blood transfusion, need for anti-hypertensive agents (urapidil, nicardipine), need for norepinephrine and dobutamine, time to extubation (hours), total dose of morphine (mg) during the first 48 postoperative hours, type and dose of analgesic drugs, and the length of stay in the ICU and hospital. The length of stay in the ICU was defined as the number of days spent in the ICU, and the length of stay at hospital was defined as the number of days between hospital admission and hospital discharge. Chronic pain assessment was performed with the DN4 questionnaire 3 months after hospital discharge.

### Randomization

On the day of the intervention, patients were randomized to the standard group or the pupillary pain index group (CleanWeb Clinical Trial Management System, Telemedicine technologies) to achieve a ratio of 1: 1. Randomization was stratified on Euroscore2. Although the operative staff members who collected data could not be blinded to group assignments, much attention was given to ensuring strict blinding during the data collection and data analysis. Surgeons, ICU physicians and nurses were blinded to group allocation.

### Safety assessment and adverse events

Endpoints and adverse events were recorded by physicians affiliated to the Dijon clinical research department who were blind to group assignments. Morphine-related events were prospectively noted: nausea, vomiting, constipation, and acute respiratory failure.

### Statistical analyses

Sample size calculation was based on a previously published study and institutional database^7^. The inclusion of 42 patients was needed to demonstrate a difference of 30% in the total dose of sufentanyl for a mean dose of 80 µg (± 27) between the two groups, with a power of 90% and a two-tailed *p-*value of 0.05. We choose to include 50 patients to take into account potential withdrawals. The normality of the data distribution was assessed using the Shapiro–Wilk test. Quantitative data were expressed as means ± standard deviation or medians [interquartile range], as appropriate, and qualitative data were expressed as numbers (percentage) without the imputation of missing data. The primary endpoint was evaluated using a Mann–Whitney test. The secondary endpoints were evaluated using a Student’s t-test, Mann–Whitney test, Wilcoxon or paired Student’s t-test. The associations between the PPI and sufentanyl concentrations considering both time points were assessed using mixed linear modelling. A Bonferroni correction was used for repeated measurements. All hypothesis tests were 2-sided, and the threshold for statistical significance was set at *p* < 0.05 for primary and secondary endpoints. All analyses were performed using R Studio Version 1.0.143—2009–2016 R Studio, Inc from R version 3.5.0.

## Results

### Patients

**Figure 2:**
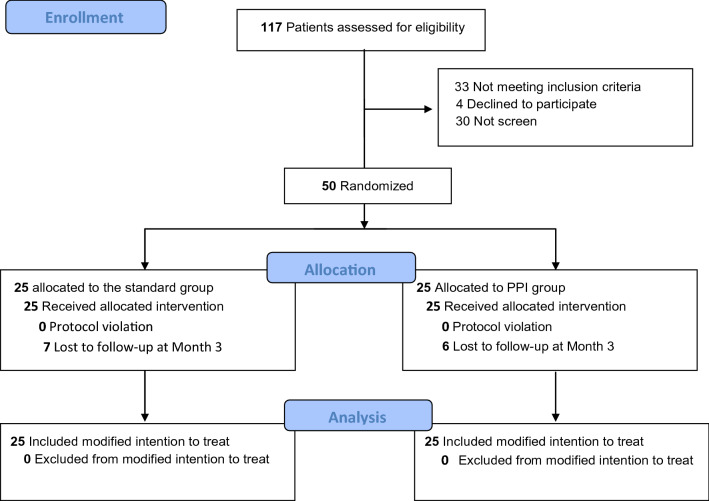
Flow chart.

Among the 87 patients screened, 50 patients were included and randomized (Fig. 2). No patients were excluded. Baselines characteristics are presented in Table 1. Median age was 67.8 (9), 88% of patients were men, and the median Euroscore II was 1.05 [0.82; 2.21] in the standard group and 1.30 [0.68; 1.73] in the PPI group.

**Table 1:** General characteristics of the study groups.

	S group (n = 25)	PPI group (n = 25)
Sex (male)	21 (84%)	23 (92%)
Age (years)	70.3 (9)	65.4 (9)
Body Mass Index (kg/m^2^)	26.4 [24.7; 29.4]	27.0 [25.3; 31.1]
**ASA, n**		
2	12 (48%)	8 (32%)
3	13 (52%)	17 (68%)
EuroSCORE II	1.05 [0.82; 2.21]	1.30 [0.68; 1.73]
Diabetes, n	9 (36%)	12 (48%)
High Blood Pressure, n	20 (83%)	18 (72%)
Dyslipidemia, n	16 (64%)	11 (44%)
Arrythmia, n	3 (12%)	6 (24%)
Left ventricular ejection fraction (%)	54 (11)	54 (11)
Chronic kidney disease, n	1 (4%)	3 (12%)
Chronic Obstructive Pulmonary Disease, n	3 (12%)	1 (4%)
**Type of surgery, n**		
Coronary artery bypass graft	19 (76%)	22 (88%)
Combined	6 (24%)	3(12%)

Data are presented as median [IQR] or n (%).

*S* standard group, *PPI* pupillary pain index group, *ASA* American Society of Anesthesiologists. All *p*-value are over 0.05.

### Primary outcome

The total dose of sufentanyl was lower in the PPI group than in the standard group: 55.8 µg [39.7–95.2] vs 83.9 µg [64.1–107.0] (p = 0.04) (Table 2).

**Table 2:** Operating characteristics and primary outcomes.

	S group (n = 25)	PPI group (n = 25)	*p*-value
Sufentanyl (µg)	83.9 [64.1; 107.0]	55.8 [39.7; 95.2]	0.04
Sufentanyl (µg/kg/h)	0.24 [0.19; 0.33]	0.17 [0.12; 0.26]	0.05
Propofol (mg)	1596 [1332; 2000]	1649 [1410; 2020]	0.62
Ketamine (mg)	25 (100%)	25 (100%)	1
Surgery duration (min)	250 (47)	243 (38)	0.53
Anesthesia duration (min)	308 (50.0)	306 (36.6)	0.88
Cardiopulmonary bypass duration (min)	115 (37)	101 (24)	0.12
Aortic cross clamp duration (min)	85 (27)	78 (21)	0.30
Time to extubation (min)	138 [111; 167]	122 [85; 151]	0.55
**Antihypertensive agent, n**			
Urapidil	11 (44%)	15 (60%)	0.26
Nicardipine	2 (4%)	4 (16%)	0.66
Atropine, n	1 (4%)	0 (0%)	1.00
**Vasoactive drugs, n**			
Ephedrine, n	17 (68%)	16 (64%)	1.00
Dose (mg)	9 [0; 19]	9 [0; 21]	0.98
Phenylephrine, n	3 (12%)	5 (20%)	0.70
Dose (µg)	0 [0; 0]	0 [0; 0]	0.44
Norepinephrine, n	14 (56%)	19 (76%)	0.14
Maximal dose (µg/kg/min)	0.11 [0; 0.22]	0.12 [0.01; 0.27]	0.21
Epinephrine, n	1 (4%)	0 (0%)	1.00
Maximal dose (µg/kg/min)	0 [0; 0]	0 [0; 0]	0.34
Dobutamine, n	3 (12%)	3 (12%)	1.00
Maximal dose (µg/kg/min)	0 [0; 0]	0 [0; 0]	0.92

Data are presented as median [IQR] or n (%).

### Secondary outcomes

**Figure 3:**
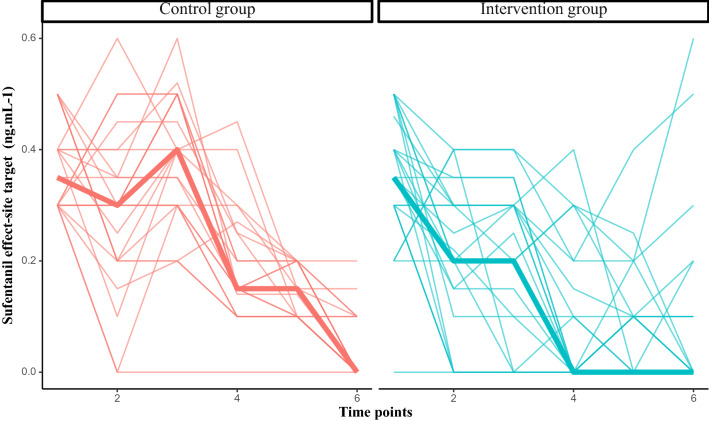
Evolution of sufentanyl site target. Time points: 2 min before orotracheal intubation (T1) 2 min before skin incision (T2); after sternotomy (T3); at the start of the CPB (T4); at CPB weaning (T5); at skin closure (T6).

The individual variations in cumulative sufentanyl dose and sufentanyl site target concentrations are presented in Fig. 3 and the Supplementary Information. Propofol consumption was not different between groups (1596 [1332–2000] vs. 1649 [1410–2020] mg; p < 0.62). BIS values, mean arterial pressure, heart rate and propofol site target concentrations did not differ between the two groups either (Supplementary Information).

The per-operative administration (during anesthesia and CPB) of antihypertensive medications (urapidil and nicardipine), inotropic and vasopressor agents did not differ between the two groups (Table 2). The troponin peak was not significantly different between the two groups (6.4 ng/ml [3.6–9] vs 4.8 ng/ml [3.1–7.5], p = 0.27).

The cumulative dose of morphine (mg) was not significantly different between groups (23 mg [15–53] vs 24 mg [17–46]; p = 0.95), nor at any time points. The use of complementary rescue pain killers did not differ between the two groups (48% vs 32% p = 0.25). There were no differences in VAS at any of the time-points (at extubation [H0], H6, H24, and H48) or in ICU and hospital lengths of stay (Table 3).

**Table 3:** Post-operative course of secondary outcomes.

	S group (n = 25)	PPI group (n = 25)	*p*-value
Total postoperative morphine consumption at 48 h (mg)	23 [15; 53]	24 [17; 46]	0.95
**Additional analgesics, n**			
Tramadol	3 (12%)	4 (16%)	
Nefopam	8 (32%)	1 (4%)	0.25
Ketoprofen	4 (16%)	5 (20%)	
**Static VAS**			
H0	5 [3; 5]	2 [0; 5]	0.29
H6	1 [0; 3]	2 [0; 3]	1.00
H24	2 [0; 3]	1 [0; 3]	1.00
H48	0 [0; 0]	0 [0; 1]	1.00
**Dynamic VAS**			
H0	5 [3; 7]	5 [0; 6]	1.00
H6	3 [2; 5]	3 [2; 4]	1.00
H24	4 [3; 7]	4 [2; 5]	0.69
H48	2 [0; 3]	2 [0; 3]	1.00
Troponin Ic (ng/ml)	6.4 [3.6–9.0]	4.8 [3.1–7.5]	0.27
**Length of stay**			
ICU (h)	37 [21; 72]	46 [22; 84]	0.99
Hospital (day)	8 [7; 9]	8 [7; 9]	0.91
Chronic pain	0 (0%)	2 (10%)	0.49
Morphine adverse effects	8 (32%)	7 (28%)	
Nausea and vomiting	5 (20%)	3 (12%)	
Constipation	3 (12%)	2 (8%)	1.00
AUR	0 (0%)	2 (8%)	
Confusion	3 (12%)	2 (8%)	
**Respiratory events**			
Acute respiratory failure	2 (8%)	1 (4%)	1.00
Reintubation	1 (4%)	1 (4%)	1.00
Arrhythmia	7 (28%)	9 (36%)	0.54

Data are presented as median [IQR] or n (%). H0 refers to tracheal extubation.

*VAS* visual analog scale, *ICU* intensive care unit, *AUR* acute urinary retention.

There was no difference in the incidence of morphine related digestive adverse event (8 (32%) vs 7 (21%), p = 1) or respiratory events (2 (8%) vs 1 (4%), p = 1). We found no significant difference in the prevalence of chronic pain at 3 months: standard group n = 0 (0%) vs PPI group n = 2 (10%) p = 0.49.

## Discussion

The present results demonstrate that the administration of sufentanyl based on the PPI algorithm decreased the total dose consumed during cardiac surgery. The decreased use of sufentanyl was not associated with higher operative hemodynamic instability, increased use of hypnotic agents, higher postoperative analgesia requirement (opioid and non-opioid analgesia), more severe pain, or lower hyperalgesia phenomenon at 3 months.

Despite several publications demonstrating the usefulness of pupillometry dilation to track nociception during anesthesia, few physicians use this technology regularly. Sabourdin et al.demonstrated that the use of pupillometry decrease the need for intraoperative remifentanil during major gynecological surgery ^13^. These authors described the challenge in defining a relevant pupil dilatation threshold to assess analgesia. In this context, the manufacturer has developed a PPI that provides an “analgesia score” informing the physician of the status of the nociception/antinociception balance. Wildemeersch et al.and Vide et al.have validated the use of the PPI during general anesthesia with remifentanil ^14, 20^. The PPI is able to provide information on pupillary dilatation reflex associated with standardized nociceptive stimulus. A PPI score lower than 4 was demonstrated to be associated with suppression of response to nociceptive stimulus. Later, Sabourdin et al. demonstrated that the PPI decreases with a single dose of alfentanil, confirming its ability to track nociceptive/antinociceptive response to the increase of analgesia level^15^. Finally, the present study demonstrats the ability of an algorithm based on the PPI to titrate the opioid dose during anesthesia.

We specifically choose cardiac surgery because the surgical conditions are associated with several factors that affect pharmacokinetics and the effects of opioids (age, cardiovascular comorbidities, chronic treatment, CPB). There are several reasons for the high doses of opioids used during cardiac surgery, such as the high level of nociceptive stimulus associated with sternotomy, the pharmacokinetic disturbance associated with CPB, and the cardioprotective effect of opioids^1^. It is well known, however, that the effects of opioids are highly variable^3^. Interestingly, the main difference between groups may have been a result of the lower sufentanyl target concentrations in the PPI group from the beginning of the CPB procedure. Because of the pharmacokinetic disturbance associated with CPB and the lack of clinical markers of nociception (heart rate, pulsatile blood pressure), physicians use to maintain sufentanyl infusion during CPB. By tailoring nociceptive/antinociceptive balance to the patient, the PPI is able to individualize analgesia treatment independently of these factors, even during CPB. Similar to Vide et al., we demonstrated a weak association between the sufentanyl site target concentrations and the PPI score, reflecting the intra-individual variability in opioid needs. Usually, physicians increase the sufentanyl dose before the pain stimulus has begun. In the PPI group, there was a different pattern of sufentanyl management (Fig. 3). At the beginning, the sufentanyl dose was decreased (before the surgical stimuli) whereas the dose was increased in the standard group. Finally, because the cumulated dose was lower throughout the surgery, the increase of the sufentanyl dose at the end of the intervention was not associated with a higher overall dose.

Despite a lower cumulative dose of sufentanyl, we did not demonstrate more hemodynamic instability or an increased need for hypnotics. The two groups were found to have similar needs for both anti-hypertensive and vasopressive agents, which is of clinical importance because arterial hypotension or hypertension may be associated with clinical outcomes such as kidney or brain injuries, particularly in cardiac surgery patients^21^. A recent study evaluating opioid-free balanced general anesthesia has been demonstrated to be associated with higher hypnotic requirements and more hemodynamic events^7^. These effects may limit the utility of an opioid-free strategy. Similarly, we did not observe a difference in postoperative peak troponin between the two groups. When taken together, these results suggest that because opioid use was tailored to individual analgesia requirements, the lower dose of sufentanyl was not associated with a higher use of hypnotic agents or hemodynamic events.

Contrary to previous studies, we found no differences in postoperative analgesia or hyperalgesia. Using a pupillometry-guided intraoperative remifentanil algorithm, Sabourdin et al. demonstrated decreased postoperative analgesia requirements and hyperalgesia^13^. In our study, we chose to administer sufentanyl via continuous infusion because it is widely used in cardiac surgery. Most previous studies have evaluated remifentanil, which is known to be associated with higher postoperative opioid requirements and hyperalgesia^21–24^. In our study, all patients received ketamine and postoperative multimodal analgesia. This strategy aims to minimize opioid consumption, side effects, and to avoid hyperalgesia^19, 25^. Our results are in line with previous findings on cardiac postoperative chronic pain^19^. Because of the multimodal analgesia strategy and the low number of included patients, we were not able to demonstrate a decrease in opioid-related side effects with the decrease of the total operative dose.

Pupillometry is not the only analgesia monitoring device available. However, the use of analgesia monitoring devices based on RR variability or plethysmography waveform analysis may be difficult during CPB^7, 26, 27^. Moreover, the frequent hemodynamic changes associated with cardiac surgery can lead to changes in these parameters in addition to the effects of analgesia.

There are several limitations to our study. Though this was a single blind study, the patients, surgeons, and ICU physicians were blinded to group allocation. There are many obstacles to designing a double-blind randomized study evaluating a therapeutic strategy, which is why we opted for single blinding. This was also a monocentric study, liming external validity. We were unable to demonstrate differences in our secondary outcomes, probably due to insufficient power. The aim of our study was to demonstrate the ability of the PPI algorithm to guide intraoperative opioid administration in cardiac surgery because there are no previous studies on this subject; larger multicentric studies will be needed to validate our results. The PPI algorithm used here was based on our clinical experience and study, but the threshold of 4 has been validated by other authors ^15^. We did not measured PPI in the control group, even though those data could have provided useful information about nociception/antinociception balance. The PPI is not used for continuous monitoring, and nociception events may therefore have been missed. However, PPI measurements were done at predefined time-points and at each hemodynamic change. Because additional analgesics were given at the discretion of the physician, it may introduce a bias for the quantification of post-operative analgesia. Therefore, a standardized multimodal analgesic regimen would have been of interest.

In conclusion, our opioid strategy based on the PPI algorithm was associated with a decrease in sufentanyl consumption during cardiac surgery. The reduction in administered opioids during surgery was not associated with higher use of hypnotics or hemodynamic instability. We did not observe a decrease in postoperative analgesia requirement or chronic pain.

## Supplementary information

Supplementary Information 1.

Supplementary Information 2.

## Data Availability

All relevant data supporting the conclusions of this article is included within the article.
