# Supplementary material for: Pupillometry pain index decreases intraoperative sufentanyl administration in cardiac surgery: a prospective randomized study
Source: Sci Rep. 2020 Dec 3;10:21056. doi: 10.1038/s41598-020-78221-5 (PMC7713228; doi:10.1038/s41598-020-78221-5)
Supplement: Supplementary file 1 — Supplementary Information 1. [file 41598_2020_78221_MOESM1_ESM.docx]

Appendix 1

# Pupillometry pain index decreases intraoperative sufentanyl administration in cardiac surgery: A prospective randomized study. Vivien Berthoud^1^; Maxime Nguyen^1,2^; Anouck Appriou^1^; Omar Ellouze^1^; Mohamed Radhouani^1^; Tiberiu Constandache^1^; Sandrine Grosjean^1^; Bastien Durand^1^; Isabelle Gounot^1^, Pierre-Alain Bahr^1^; Audrey Martin^1^; Nicolas Nowobilski^1^; Belaid Bouhemad^1,2^; Pierre-Grégoire Guinot^1,2^.

^1^Anaesthesiology and Critical Care Department, Dijon University Hospital, 2 Bd Maréchal de Lattre de Tassigny, F-21000 Dijon, France.

^2^ Univ. Bourgogne Franche-Comté, LNC UMR1231, F-21000 Dijon, France ; INSERM, LNC UMR1231, F-21000 Dijon, France ; FCS Bourgogne-Franche Comté, LipSTIC LabEx, F-21000 Dijon, France.

Sufentanil evolution

|  | S group (n=25) | PPI group (n=25) | ***p*-value** |
| --- | --- | --- | --- |
| Dose sufentanil TX to TX + 1 (ug/kg) | | |  |
| <T1 | 0.24 [0.21;0.32] | 0.28 [0.22;0.37] | 1.00 |
| T1-T2 | 0.16 [0.11;0.21] | 0.13 [0.06;0.23] | 1.00 |
| T2-T3 | 0.07 [0.01;0.14] | 0.01 [0.00;0.04] | 0.04 |
| T3-T4 | 0.21 [0.11;0.37] | 0.06 [0.00;0.25] | 0.08 |
| T4-T5 | 0.18 [0.09;0.28] | 0.06 [0.00;0.20] | 0.16 |
| T5-T6 | 0.06 [0.03;0.14] | 0.03 [0.00;0.15] | 1.00 |
| > T6 | 0.00 [0.00;0.00] | 0.00 [0.00;0.00] | 1.00 |
| Sufentanil site-target (ng/ml) |  |  |  |
| T1 | 0.35 [0.30;0.40] | 0.35 [0.30;0.46] | 1.00 |
| T2 | 0.30 [0.20;0.35] | 0.20 [0.10;0.30] | 0.31 |
| T3 | 0.40 [0.30;0.50] | 0.20 [0.00;0.30] | <0.01 |
| T4 | 0.15 [0.15;0.25] | 0.00 [0.00;0.15] | <0.01 |
| T5 | 0.15 [0.10;0.20] | 0.00 [0.00;0.10] | 0.01 |
| T6 | 0.00 [0.00;0.10] | 0.00 [0.00;0.10] | 1.00 |

*p*-values refers to between group comparisons. Time points: 2 minutes before orotracheal intubation (T1) 2 minutes before skin incision (T2); after sternotomy (T3); at the start of the CPB (T4); at CPB weaning (T5); at skin closure (T6)

Appendix 2

Hypnotic

|  | S group (n=25) | PPI group (n=25) | ***p*-value** |
| --- | --- | --- | --- |
| Propofol effect-site (ug/ml) |  |  |  |
| T1 | 4.50 [4.00;5.00] | 4.50 [4.00;5.62] | 1 |
| T2 | 2.50 [2.00;3.10] | 2.75 [1.98;3.00] | 1 |
| T3 | 3.00 [2.00;3.43] | 2.75 [2.00;3.13] | 1 |
| T4 | 2.00 [1.50;2.40] | 2.00 [2.00;2.50] | 1 |
| T5 | 2.00 [1.50;2.20] | 2.10 [1.80;2.50] | 1 |
| T6 | 2.00 [1.50;2.20] | 2.00 [1.80;2.30] | 1 |
| BIS® (unite) |  |  |  |
| T1 | 49.4 (15.1) | 47.6 (14.8) | 1 |
| T2 | 49.9 (9.32) | 47.1 (10.7) | 1 |
| T3 | 46.8 (9.94) | 41.8 (9.50) | 0.47 |
| T4 | 42.3 (4.97) | 42.1 (6.75) | 1 |
| T5 | 45.8 (8.65) | 45.7 (10.15) | 1 |
| T6 | 47.2 (7.76) | 46.6 (9.10) | 1 |

*p*-values refers to between group comparisons. Time points: 2 minutes before orotracheal I ntubation (T1) 2 minutes before skin incision (T2); after sternotomy (T3); at the start of the CPB (T4); at CPB weaning (T5); at skin closure (T6)

Appendix 3.

Pupillometry pain index evolution

| PPI | PPI group (n=25) |
| --- | --- |
| T1 | 5.00 [4.00;6.00] |
| T2 | 2.00 [1.00;5.25] |
| T3 | 2.00 [1.00;2.25] |
| T4 | 2.00 [1.75;6.00] |
| T5 | 5.00 [2.00;7.00] |
| T6 | 3.00 [2.00;5.00] |

PPI: Pupillometry pain index. Time points: 2 minutes before orotracheal intubation (T1) 2 minutes before skin incision (T2); after sternotomy (T3); at the start of the CPB (T4); at CPB weaning (T5); at skin closure (T6)
