# Supplementary material for: Pupillometry pain index decreases intraoperative sufentanyl administration in cardiac surgery: a prospective randomized study
Source: Sci Rep. 2020 Dec 3;10:21056. doi: 10.1038/s41598-020-78221-5 (PMC7713228; doi:10.1038/s41598-020-78221-5)
Supplement: Supplementary file 2 — Supplementary Information 2. [file 41598_2020_78221_MOESM2_ESM.docx]

**SUPPLEMENTARY MATERIAL**

# **Pupillometry pain index decreases intraoperative sufentanyl administration in cardiac surgery: A prospective randomized study.** Vivien Berthoud^1^; Maxime Nguyen^1,2^; Anouck Appriou^1^; Omar Ellouze^1^; Mohamed Radhouani^1^; Tiberiu Constandache^1^; Sandrine Grosjean^1^; Bastien Durand^1^; Isabelle Gounot^1^, Pierre-Alain Bahr^1^; Audrey Martin^1^**;** Nicolas Nowobilski^1^; Belaid Bouhemad^1,2^; Pierre-Grégoire Guinot^1,2^.

^1^Anaesthesiology and Critical Care Department, Dijon University Hospital, 2 Bd Maréchal de Lattre de Tassigny, F-21000 Dijon, France.

^2^ Univ. Bourgogne Franche-Comté, LNC UMR1231, F-21000 Dijon, France ; INSERM, LNC UMR1231, F-21000 Dijon, France ; FCS Bourgogne-Franche Comté, LipSTIC LabEx, F-21000 Dijon, France.

**Institutional cardiopulmonary bypass management**

Cardiopulmonary bypass was conducted with a heart-lung machine (Stockert Sorin S5 Heart Lung, Milan, Italy) at a target blood flow of 2.4 l min/m. The mean arterial blood pressure (MAP) was maintained at more than 65 mmHg by increasing the pump flow rate or, if required, by administering a bolus of norepinephrine (5μg). The CPB circuit was primed with 1500 ml of crystalloids (Plasma-Lyte®; Baxter, Lessines, Belgium) and 5000 ui of heparin. After systemic heparinization (300 ui/kg) to obtain a hemochron level of 400 s, median sternotomy was performed and aortic and right auricular cannulations were started. Normoglycemia was maintain using intravenous insulin (intravenous bolus of 5–10 ui) if necessary. Patients with a hemoglobin value below 8 g/dlreceived homologous red blood cell transfusions. Heparin was reversed with protamine at a 1:1 ratio.

Before performing the study, we measured the intra-operator coefficients of variation and the intra-class correlation coefficient of the PPI measurement during the CPB to ensure the feasibility and availability of the PPI. The median variation coefficient was 9 [0 to 12] %, and the intra-class correlation coefficient was 1.00 [0.99;1.00].


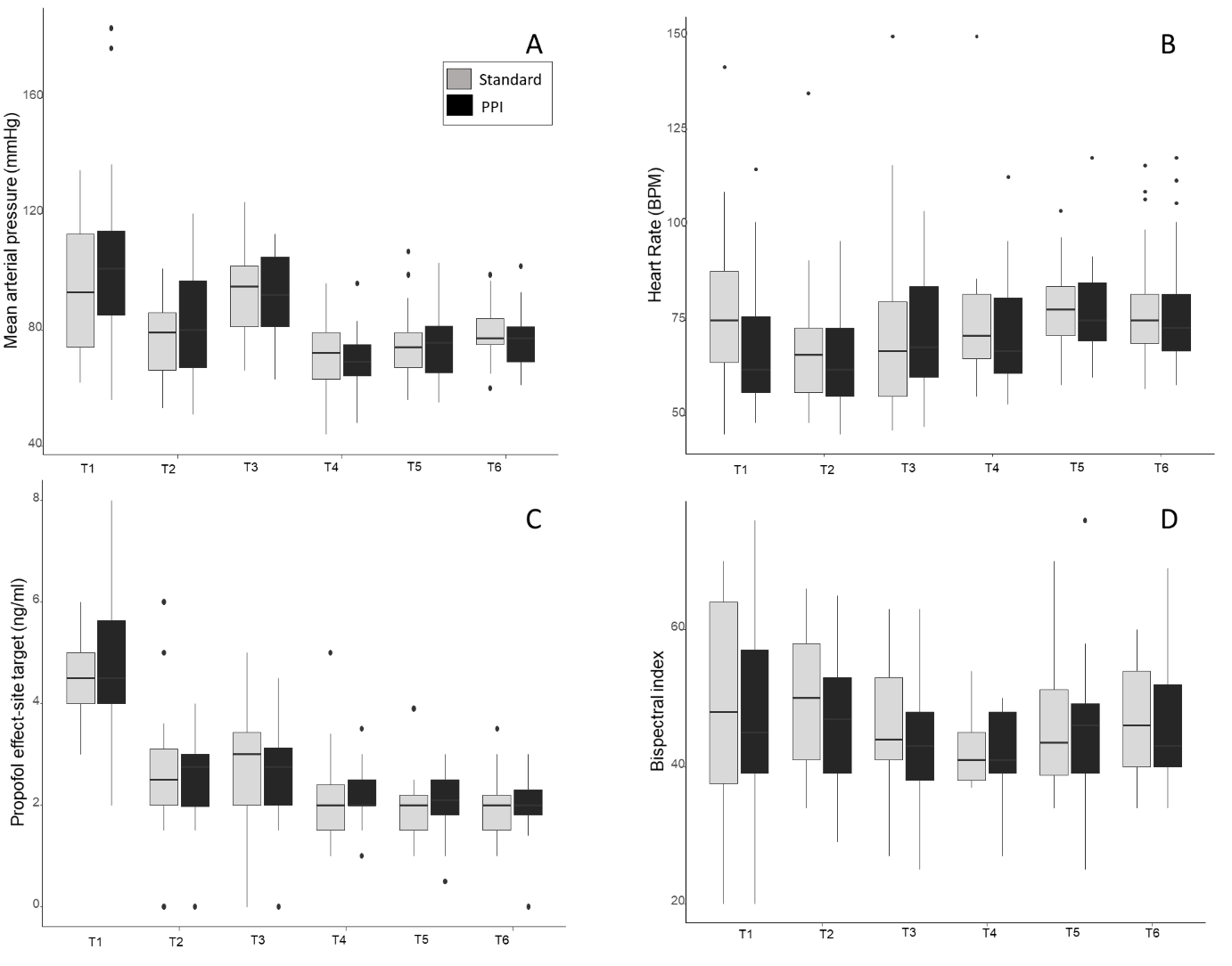


**Figure 1. Evolution of mean arterial pressure (A), Heart rate (B), propofol effect-site concentration (C) and Bispectral index (D) during surgery, according to group allocation.**

PPI: pupillometry pain index

Time points: 2 minutes before orotracheal intubation (T1) 2 minutes before skin incision (T2); after sternotomy (T3); at the start of the CPB (T4); at CPB weaning (T5); at skin closure (T6)

**
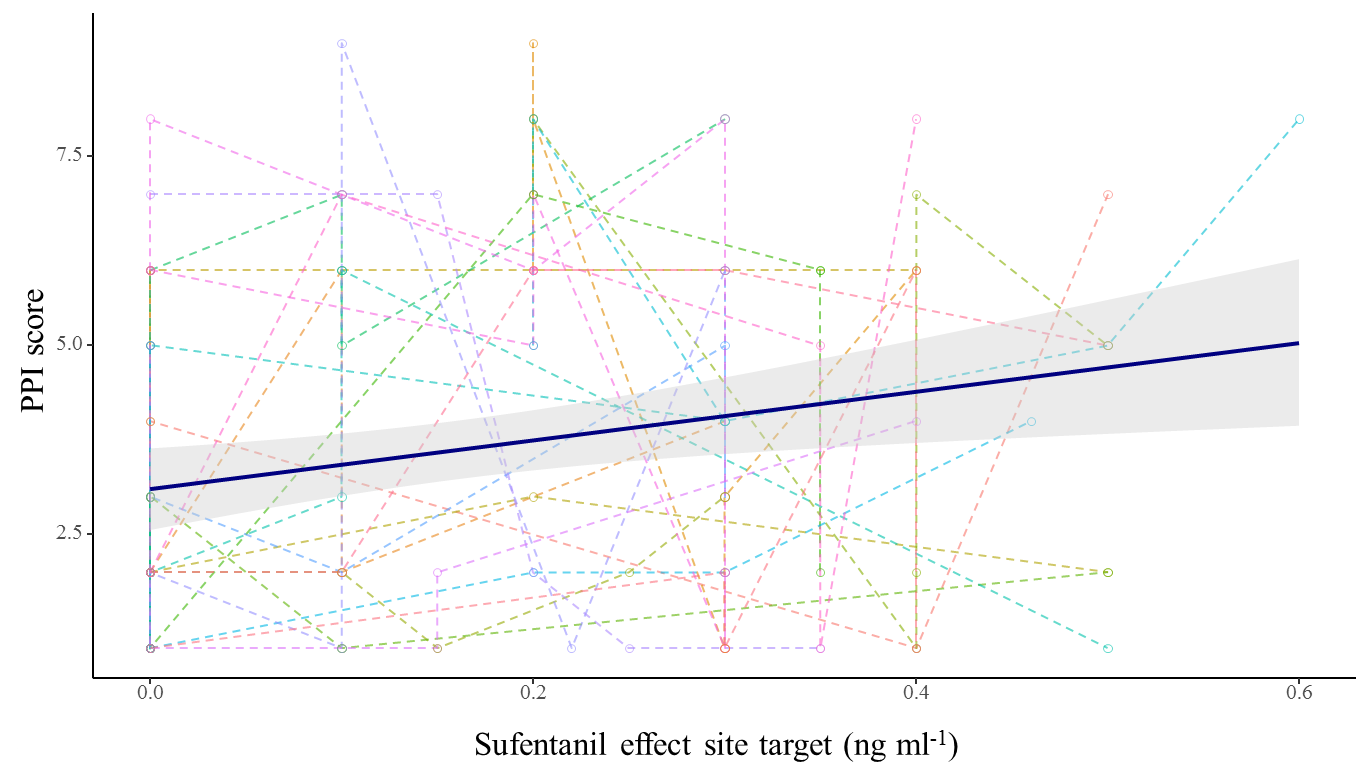
**

**Figure 2. Correlation between sufentanil effect-site concentration and PPI in the intervention group.** The dashed line links data from the same patient. The plain line is the regression line using all measurements, with the grey zone representing the 95% confidence interval. PPI and Sufentanil target site concentration were associated (regression coefficient = 3, standard error = 1.5, p = 0.04). Assessment of association was performed using a mixed linear model (time was added as a fixed effect and patients were used as random intercepts).


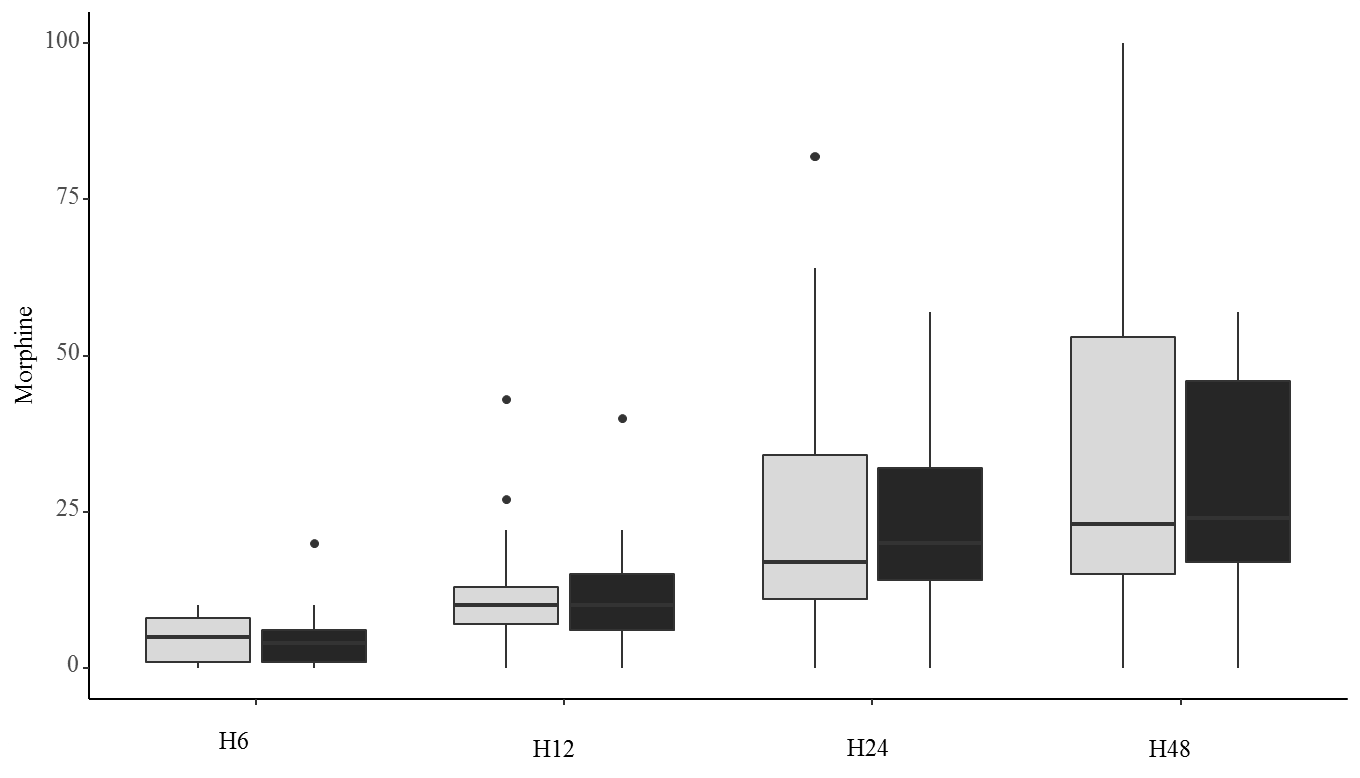


**Figure 3. Cumulative morphine dose at different post-operative time points**


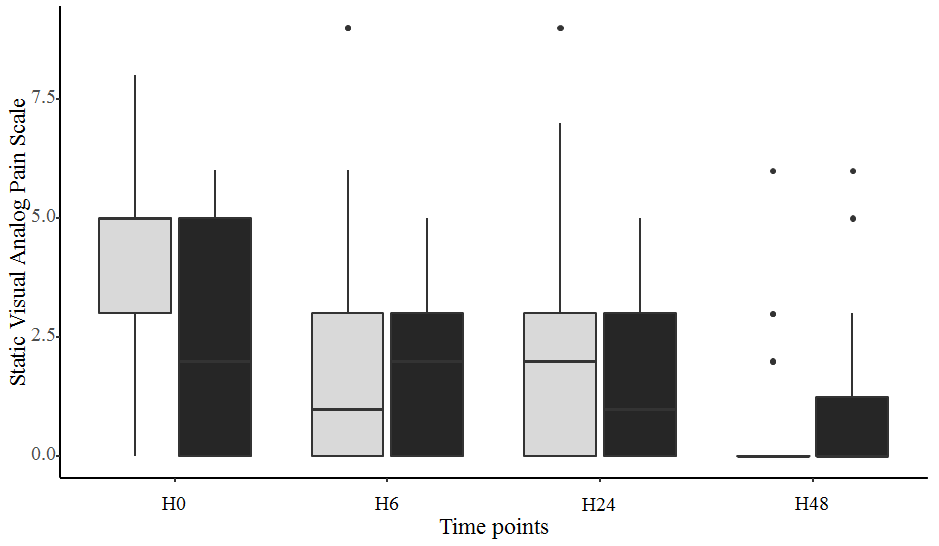

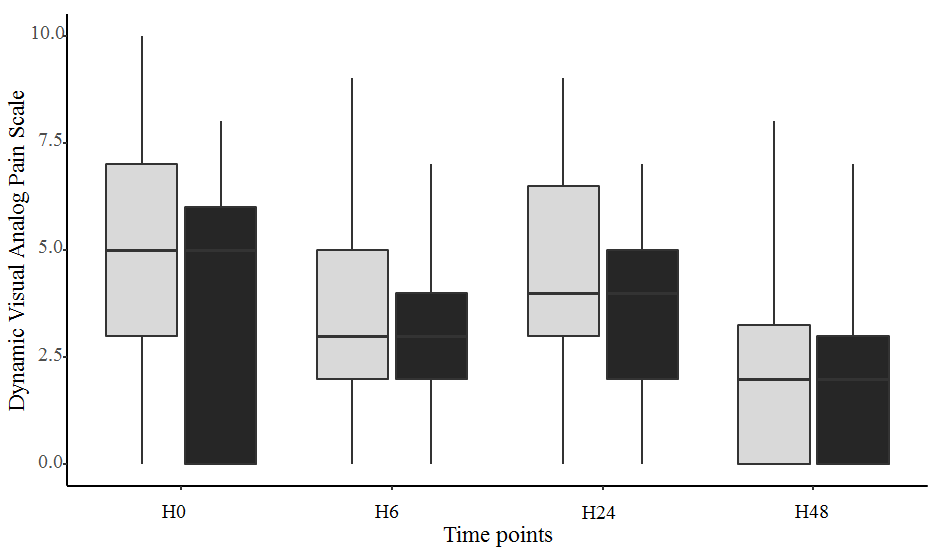


B

A

**Figure 4. Visual analog pain scale at rest (A) and during effort (B) at different post-operative time points**

Appendix 1

Change in sufentanil dose

|  | Standard group (n=25) | PPI group (n=25) | ***p*-value** |
| --- | --- | --- | --- |
| Dose sufentanil TX to TX + 1 (ug) |  |  |  |
| <T1 | 20.0 [17.4;25.0] | 23.6 [20.1;26.4] | 0.30 |
| T1-T2 | 12.5 [9.57;19.6] | 11.0 [6.00;19.0] | 1.00 |
| T2-T3 | 5.00 [1.00;11.7] | 1.00 [0.00;3.35] | 0.04 |
| T3-T4 | 15.4 [9.95;32.6] | 3.50 [0.00;23.1] | 0.11 |
| T4-T5 | 15.3 [10.5;20.0] | 4.50 [0.00;17.4] | 0.32 |
| T5-T6 | 5.20 [2.87;9.65] | 2.45 [0.00;10.3] | 1.00 |
| >T6  Sufentanil site-target (ng/ml) | 0.00 [0.00;0.00] | 0.00 [0.00;0.00] | 1.00 |
| T1 | 0.35 [0.30;0.40] | 0.35 [0.30;0.46] | 1.00 |
| T2 | 0.30 [0.20;0.35] | 0.20 [0.10;0.30] | 0.31 |
| T3 | 0.40 [0.30;0.50] | 0.20 [0.00;0.30] | <0.01 |
| T4 | 0.15 [0.15;0.25] | 0.00 [0.00;0.15] | <0.01 |
| T5 | 0.15 [0.10;0.20] | 0.00 [0.00;0.10] | 0.01 |
| T6 | 0.00 [0.00;0.10] | 0.00 [0.00;0.10] | 1.00 |

*p*-values refers to between-group comparisons. Time points: 2 minutes before orotracheal intubation (T1) 2 minutes before skin incision (T2); after sternotomy (T3); at the start of the CPB (T4); at CPB weaning (T5); at skin closure (T6)

Appendix 2

Hypnotics

|  | Standard group (n=25) | PPI group (n=25) | ***p*-value** |
| --- | --- | --- | --- |
| Propofol effect-site (ug/ml) |  |  |  |
| T1 | 4.50 [4.00;5.00] | 4.50 [4.00;5.62] | 1 |
| T2 | 2.50 [2.00;3.10] | 2.75 [1.98;3.00] | 1 |
| T3 | 3.00 [2.00;3.43] | 2.75 [2.00;3.13] | 1 |
| T4 | 2.00 [1.50;2.40] | 2.00 [2.00;2.50] | 1 |
| T5 | 2.00 [1.50;2.20] | 2.10 [1.80;2.50] | 1 |
| T6 | 2.00 [1.50;2.20] | 2.00 [1.80;2.30] | 1 |
| BIS® (unite) |  |  |  |
| T1 | 49.4 (15.1) | 47.6 (14.8) | 1 |
| T2 | 49.9 (9.32) | 47.1 (10.7) | 1 |
| T3 | 46.8 (9.94) | 41.8 (9.50) | 0.47 |
| T4 | 42.3 (4.97) | 42.1 (6.75) | 1 |
| T5 | 45.8 (8.65) | 45.7 (10.15) | 1 |
| T6 | 47.2 (7.76) | 46.6 (9.10) | 1 |

*p*-values refers to between group comparisons. Time points: 2 minutes before orotracheal intubation (T1) 2 minutes before skin incision (T2); after sternotomy (T3); at the start of the CPB (T4); at CPB weaning (T5); at skin closure (T6)
